# Long non-coding RNA LOC554202 promotes acquired gefitinib resistance in non-small cell lung cancer through upregulating miR-31 expression

**DOI:** 10.7150/jca.35097

**Published:** 2019-10-15

**Authors:** Jing He, Shidai Jin, Wei Zhang, Deqin Wu, Jun Li, Jing Xu, Wen Gao

**Affiliations:** 1 Department of Oncology, The First Affiliated Hospital of Nanjing Medical University, 300 Guangzhou Road, Nanjing, 210029, China; 2 Department of Radiology, The First Affiliated Hospital of Nanjing Medical University, 300 Guangzhou Road, Nanjing, 210029, China; 3 Department of Pharmacy, The First Affiliated Hospital of Nanjing Medical University, 300 Guangzhou Road, Nanjing, 210029, China

**Keywords:** non-small-cell lung cancer, resistance, gefitinib, LOC554202, miR-31, proliferation, RAF-MEK-ERK, PI3K-AKT

## Abstract

Non-small-cell lung cancer (NSCLC) patients with epidermal growth factor receptor (EGFR) mutation inevitably have a relapse due to the occurrence of acquired resistance, resulting in treatment failure. However, little is known about the mechanisms of acquired resistance of NSCLC patients. Here, we elucidated the expression pattern of LOC554202 and miR-31, and their biological functions and mechanisms in NSCLC with acquired EGFR TKI resistance to gefitinib. In the present study, we observed that LOC554202 and miR-31 promoted proliferation and clonogenic growth of gefitinib-resistant NSCLC cells *in vitro*. LOC554202 upregulated miR-31 expression and they both reduced sensitivity of NSCLC cells to gefitinib. In a xenograft mice model, we found that knockdown of miR-31 significantly repressed gefitinib-resistant NSCLC cells growth *in vivo*. Furthermore, both LOC554202 and miR-31 levels were significantly increased in NSCLC patients acquiring resistance to gefitinib, and the expression of LOC554202 was positively correlated with the expression of miR-31. By luciferase reporter assays, we identified RAS P21 Protein Activator 1 (RASA1) and Hypoxia Inducible Factor 1 Subunit Alpha Inhibitor (FIH-1) as direct targets of miR-31 in NSCLC cells. Mechanistically, miR-31 directly repressed RASA1 and FIH-1 expression, and thus, at least partially activated the RAF-MEK-ERK and PI3K-AKT signaling pathways in NSCLC with acquired resistance to gefitinib. In conclusion, these data will help us develop potential therapeutic targets for the diagnosis and treatment of acquired EGFR TKI resistance in EGFR-mutant NSCLC.

## Introduction

Non-small-cell lung cancer (NSCLC) accounts for approximately 85% ~ 90% of primary lung cancer. However, about two-thirds of the patients were diagnosed at the advanced stage of NSCLC^1^, ^2^. In recent years, molecular target-based therapies targeting epidermal growth factor receptor (EGFR) tyrosine kinase inhibitors (TKIs) have brought new hope for NSCLC patients, especially for those acquired with EGFR-sensitive mutations^3^. Despite impressive initial response, acquired drug resistance inevitably have a relapse in almost all patients, resulting in treatment failure^4^, ^5^. However, the mechanisms of acquired resistance of NSCLC patients remain unknown. Therefore, exploring the molecular mechanism of EGFR-TKIs resistance will help us better improve the efficacy of targeted drug therapy.

Long noncoding RNAs (lncRNAs) represent a category of noncoding RNAs, which are longer than 200 nucleotides in length and cannot be translated into proteins (or limited protein-coding capacity)^6^, ^7^. In recent years, a large amount of reports suggested that lncRNAs played crucial roles in a large variety of biological processes, such as cell differentiation, carcinogenesis and cancer progression, protein synthesis, mRNA splicing, gene expression/ regulation, signal transduction, migration/invasion and metabolism etc.^8^, ^9^. Deregulated lncRNAs were reported in a variety of malignant tumors including liver cancer^10^, gastric cancer^11^, ^12^, bladder cancer 13, breast cancer^14^, osteosarcoma^15^, glioblastoma 16, colorectal cancer 17, 18 and also NSCLC^19^, ^20^, which suggested that lncRNAs were associated with tumorigenesis, cancer progression and response to therapy^21^, ^22^.

As another kind of noncoding RNAs, microRNAs (miRNAs) (18 ~ 24 nucleotides in length) also attract growing interest among the scientific community, which could inhibit gene expression by directly targeting the 3'-untranslated region (3'-UTR) of target gene mRNAs^23^. MiRNAs are becoming promising therapeutic targets for cancer treatment because of their important biological functions^24^, ^25^. Notably, the roles of miRNAs in acquired resistance of NSCLC have been reported. For example, Li and Shen *et al.* reported that miR-21 overexpression is associated with acquired resistance of EGFR-TKI by regulating PTEN in NSCLC^26^, ^27^. Yue *et al.* demonstrated that epigenetic knockdown of miR-483-3p could promote acquired gefitinib resistance by regulating integrin β3 in NSCLC^28^. Therefore, miRNAs seem to be promising therapeutic targets for treatment of NSCLC patients who acquire resistance to EGFR TKI treatment.

LncRNA loc554202 (LOC554202) is located on human chromosome 9 and was reported deregulated in different types of cancers, suggesting that LOC554202 may play as oncogene or anti-oncogene in these cancers^29^, ^30^. Reports suggested that LOC554202 is the host gene of miR-31 and its expression was correlated with LOC554202 in tumors^31^. In NSCLC, although miR-31 has been proved deregulated, the roles of miR-31 in NSCLC patients who acquire resistance to EGFR TKI treatment were still largely unclear. In this study, we aimed to explore the expression patterns and functions of LOC554202 and miR-31 in NSCLC acquired resistance to gefitinib, which will help us develop a potential therapeutic target in the treatment of patients with NSCLC.

## Materials and Methods

### Patient samples, cell culture, transfection and lentivirus infection

Plasma samples were collected from NSCLC patients with EGFR-TKI treatment failure who were hospitalized in the First Affiliated Hospital of Nanjing Medical University. IIIB or IV stage NSCLC patients with common EGFR mutation (exon 19 deletion and L858R) treated with first generation of EGFR-TKIs between June 2015 to May 2018. EGFR-mutant NSCLC patients who had a history of disease progression after EGFR-TKIs therapy available plasma at the time points of before and after gefitinib treatment (n = 11). The basic clinical parameters of the enrolled patients were shown in Supplementary Table 1. All NSCLC patients provided written informed consent and our study was approved by the Ethics Committee of the First Affiliated Hospital of Nanjing Medical University.

### Cell culture, transfection and lentivirus infection

Human PC9 and HCC827 cells were cultured with DMEM (Gibco) supplemented with 10% fetal bovine serum (FBS; Gibco). Gefitinib resistant cell lines PC9GR and HCC827GR were fabricated according to a previous report^31^. Before *in vitro* experiments, PC9GR and HCC827GR cells were cultured in 2 μM gefitintib medium for 4 ~ 5 days to confirm the resistance to gefitinib. For transient transfection, miR-31, miR-NC, miR-31-inhibitors, inhibitor-NC and siRNA mimics (GenePharma, Shanghai, China) were transfected with Lipofectamine 3000 (Invitrogen) according to manufacturer's instructions. To fabricate stable PC9GR cells expressing miR-31 inhibitors and NC controls, lentivirus were fabricated in 293T cells with pLKO.1-puro plasmids containing miR-31 inhibitors (Anti-miR-31) or negative controls (Anti-NC). Lentivirus and 2.5 μg/mL Polybrene (Yeasen; 40804ES76) were mixed to infect PC9GR cells. Positive PC9GR cells stably expressing miR-31 inhibitors and NC controls were selected by 1 μg/mL puromycin (Beyotime, ST551; China).

### Real-time PCR analysis

Total RNA was isolated from cells (PC9/PC9GR and HCC827/HCC827GR), xenograft tumor tissues and plasma from patients using TRIzol reagent (Invitrogen, USA). RNA was transcribed to cDNA by a HiScript II Reverse Transcriptase kit (R201-01; Vazyme biotech co., ltd. China). Reverse transcription of miR-31 and the detection of mature miR-31 expression were performed using the miRNA reverse transcription and quantitative real-time PCR detection kit (GenePharma, Shanghai, China). GAPDH and U6 snRNA were used to normalize the expression levels of LOC554202 and miR-31, respectively. The primer sequences were: GAPDH, F: 5'-ACCCACTCCTCCACCTTTGA-3'; R: 5'-CTGTTGCTGTAGCCAAATTCGT-3'. LOC554202, F: 5'-TCTCTGGTGCTTCCCTCCTT-3', R: 5'-GATCTAAGCTTGAGCCCCCA-3'. The relative LOC554202 and miR-31 expression was calculated by 2^-ΔΔCt^ method.

### Luciferase reporter assays

The 3'-UTRs of RASA1 or FIH-1 (containing miR-31 binding sites) were cloned into a pMIR-luciferase reporter plasmid (Promega). The mutation of miR-31 binding sites in 3'-UTRs of RASA1 (UCUUGCC was mutated to UGUUCGG) or FIH-1 (UCUUGCC was mutated to UGUUCGG) was performed by a Muta Direct Site-directed Mutagenesis kit (SDM-15; Beijing SBS Genetech Co, Ltd, Beijing, China). Efficient mutation of miR-31 binding sites in 3'-UTRs of RASA1 or FIH-1 was confirmed by further DNA sequencing. Luciferase activities were analyzed using the luciferase reporter gene system (Promega) according to the manufacturer's instructions. Luciferase activity was normalized by β-galactosidase activity (β-gal). Independent triplicate experiments were performed.

### Western blot assay

Protein was extracted from PC9, PC9GR, HCC827 cells using RIPA Lysis Buffer with protease and phosphatase inhibitors (P0013B; Beyotime; China). BCA Protein Assay kit was used to determine the protein concentration (P0012S; Beyotime; China). Cell lysate was separated by SDS-PAGE electrophoresis and then transferred to PVDF membranes (Millipore). Next, the PVDF membranes was incubated with primary antibodies and followed by horseradish peroxidase-conjugated secondary antibody (Sigma) after blocking with PBST containing 5% non-fat milk. The protein signals were visualized by enhanced chemiluminescence ECL reagent (Thermo Scientific Pierce). GAPDH was used as a loading control.

### Proliferation and colony-formation assays

Cell proliferation was measured by Cell Counting Kit-8 (CCK-8) assays (Vazyme; A311-01) according to the manufacturer' instructions. 2000 ~ 4000 cells were seeded into 96-well plate. OD450 was measured at 24, 48 and 72 h after incubation with CCK-8 solutions for 2 h at 37℃. Colony-formation ability was measured by plate colony-formation assays. The same number of cells were seeded into 6-well plate. After two weeks, cells were fixed by methanol and then stained with 0.1% crystal violet for 30 min at room temperature. Lastly, cell colonies were imaged and counted (>50 cells) using a microscope.

### *In vivo* assays

Nude mice (Female; 4 ~ 6 weeks) were purchased from the Model Animal Research center of Nanjing University. To establish xenograft tumor models, 5×10^6^ gefitinib-resistant PC9GR cells overexpressing miR-31 (Anti-miR-31) inhibitors and controls (Anti-NC) in 100 μL PBS (containing 10% Matrigel) were injected subcutaneously into the left flanks of each mouse (5 mice per group). The tumor sizes were measured every five days after injection. 25 days later, all mice were euthanized and subcutaneous tumor tissues were isolated. The volume of tumor tissues was calculated as 0.5 × Length × (Width)^2^. All xenograft tumors were weighted and then performed real-time PCR assays to analyze miR-31 levels.

### Statistical analysis

Data were presented as the Mean ± SD (Standard deviation). All statistical data were performed by using SPSS 19.0 software. The significance of differences between two groups was determined by the Student's* t*-test. A P-value less than 0.05 was considered to be statistically significant. *P < 0.05, **P < 0.01.

## Results

### LOC554202 promotes proliferation and clonogenic growth of gefitinib-resistant NSCLC cells *in vitro*

**Figure 1:**
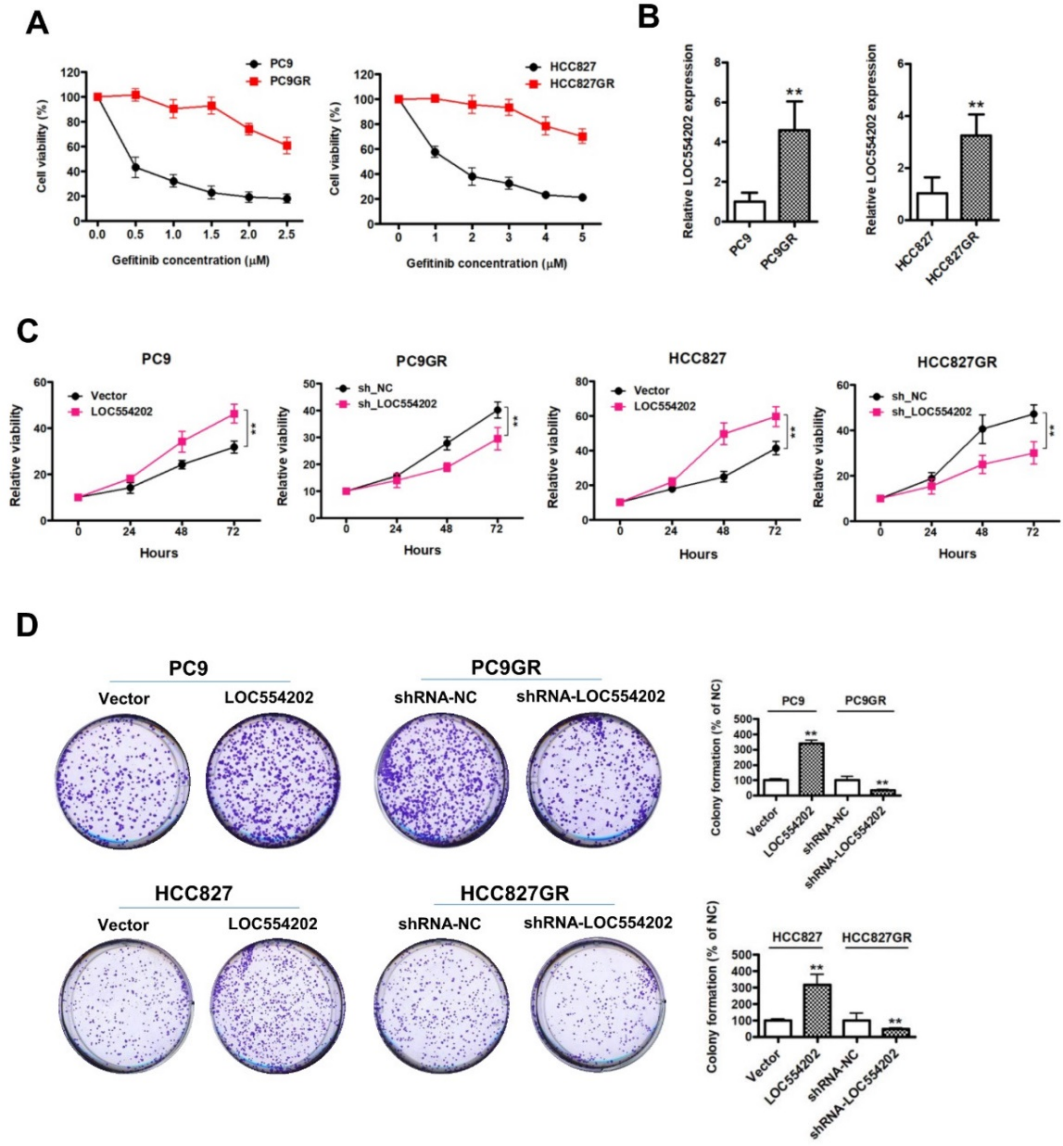
** LOC554202 promotes proliferation and clonogenic growth of gefitinib-resistant NSCLC cells *in vitro.* A**. The proliferation ability of gefitinib-sensitive (PC9 and HCC827) and gefitinib-resistant (PC9GR and HCC827GR) cells in response to different concentrations of gefitinib. **B**. Real-time PCR analysis of LOC554202 expression levels in PC9 and PC9GR (Left panel), HCC827 and HCC827GR cells (Right panel). **P < 0.01. Experiments were performed in triplicates. Data are represented as mean ± SD. **C**. Cell proliferation was measured by CCK-8 assays when LOC554202 was overexpressed in PC9 and HCC827 cells, and knocked down in PC9GR and HCC827GR cells, respectively. **P < 0.01. Experiments were performed in triplicates. Data are represented as mean ± SD. **D**. Colony-formation assays were performed when LOC554202 was overexpressed in PC9 and HCC827 cells, and knocked down in PC9GR and HCC827GR cells, respectively. **P < 0.01. Experiments were performed in triplicates. Data are represented as mean ± SD.

To explore the role of LOC554202 in acquired resistance to gefitinib, we first established* in vitro* gefitinib-resistant PC9GR and HCC827GR cell models by culturing gefitinib-sensitive NSCLC cells lines (PC9 and HCC827) with escalating doses of gefitinib. As shown in Fig. 1A, the proliferation ability of gefitinib-resistant PC9GR and HCC827GR cells was obviously increased, compared with the gefitinib-sensitive PC9 and HCC827 cells under the treatment of gefitinib. By real-time PCR assays, we found that LOC554202 was significantly upregulated in gefitinib-resistant PC9GR and HCC827GR cells in comparison with the gefitinib-sensitive PC9 and HCC827 cells (Fig. 1B; **P < 0.01), implying that LOC554202 may contribute to the acquired resistance to gefitinib. To evaluate whether LOC554202 regulates proliferation, we modulated LOC554202 levels in cells and assessed the effects on proliferation ability using CCK-8 and colony-formation assays *in vitro*. First, we observed that overexpression of LOC554202 promoted PC9 and HCC827 cells proliferation, whereas knockdown of LOC554202 decreased PC9GR and HCC827GR cells proliferation (Fig. 1C; **P < 0.01). Similarly, we also found that upregulation of LOC554202 increased PC9 and HCC827 colony-formation ability, whereas silence of LOC554202 inhibited PC9GR and HCC827GR cells clonogenic growth (In Fig. 1D; **P < 0.01). Taken together, these results suggested that LOC554202 promoted proliferation and clonogenic growth of gefitinib-resistant NSCLC cells *in vitro*.

### LOC554202 upregulates miR-31 expression and reduces sensitivity of PC9 and HCC827 cells to gefitinib

**Figure 2:**
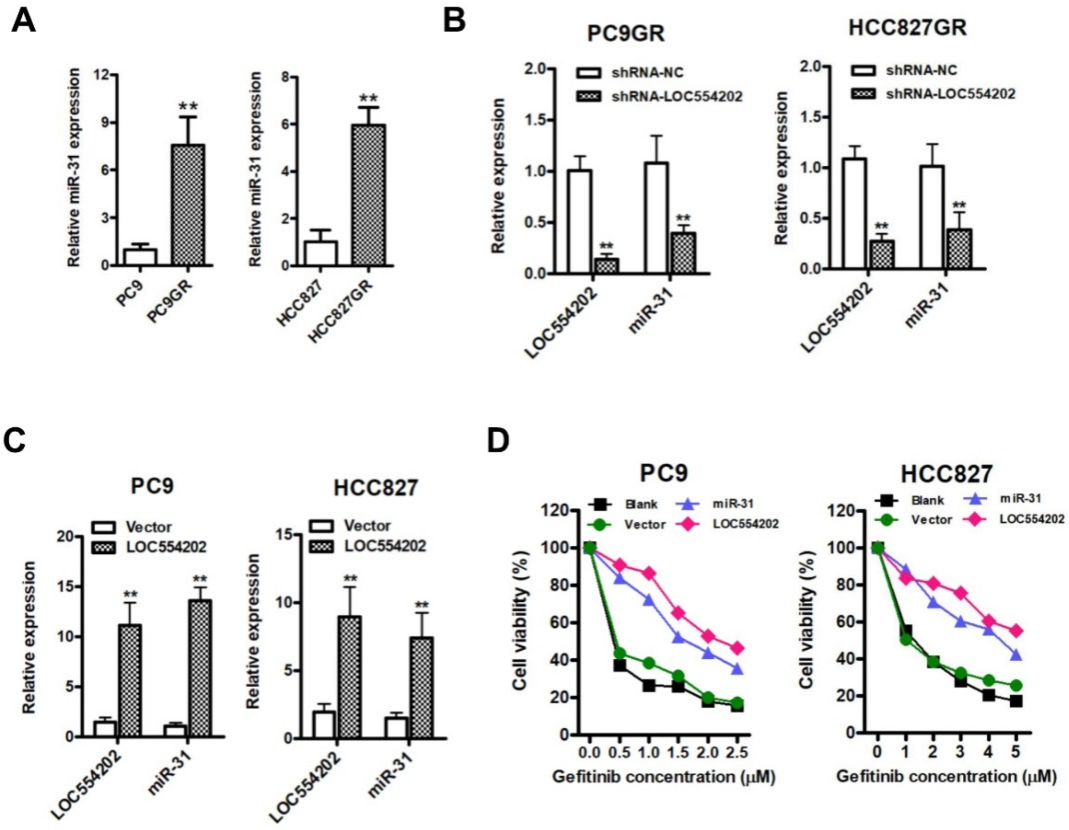
** LOC554202 upregulates miR-31 expression and reduces sensitivity of PC9 and HCC827 to gefitinib. A**. Real-time PCR analysis of miR-31 expression levels in PC9 and PC9GR (Left panel), HCC827 and HCC827GR cells (Right panel). **P < 0.01. Experiments were performed in triplicates. Data are represented as mean ± SD. **B**. Real-time PCR analysis of miR-31 expression levels in PC9GR (Left panel) and HCC827GR cells (Right panel) when LOC554202 was knocked down. **P < 0.01. Experiments were performed in triplicates. Data are represented as mean ± SD. **C**. Real-time PCR analysis of miR-31 expression levels in PC9 (Left panel) and HCC827 cells (Right panel) when LOC554202 was overexpressed. **P < 0.01. Experiments were performed in triplicates. Data are represented as mean ± SD. **D**. The sensitivity of PC9 and HCC827 cells to gefitinib was measured by CCK-8 assays when LOC554202 and miR-31 were overexpressed in PC9 (Left panel) and HCC827 (Right panel) cells, respectively. Experiments were performed in triplicates. Data are represented as mean ± SD.

Previous reports have demonstrated that miR-31 is transcribed from within the first intron of a host gene^30^, LOC554202, on human chromosome 9. The above results suggested that LOC554202 was significantly upregulated in gefitinib-resistant PC9GR and HCC827GR cells compared with the gefitinib- sensitive PC9 and HCC827 cells. Then, we detected the miR-31 levels in gefitinib-sensitive (PC9 and HCC827) and gefitinib-resistant cells (PC9GR and HCC827GR). The data from Fig. 2A clearly suggested that gefitinib-resistant PC9GR and HCC827GR cells exerted significantly higher expression levels of miR-31 than that in gefitinib-sensitive PC9 and HCC827 cells (**P < 0.01). To explore the relationship between expression levels of LOC554202 and miR-31, we manipulated the LOC554202 expression in cells and assessed the effects on the expression of miR-31. Interestingly, real-time PCR results showed that downregulation of LOC554202 reduced miR-31 expression levels in PC9GR and HCC827GR cells (Fig. 1B; **P < 0.01), whereas upregulation of LOC554202 stimulated miR-31 expression levels in PC9 and HCC827 cells (Fig. 1C; **P < 0.01), suggested that LOC554202 positively regulated miR-31 expression in NSCLC cells.

To examine whether overexpression of LOC554202 and miR-31 are associated with acquired resistance to gefitinib *in vitro*, we upregulated LOC554202 and miR-31 expression and then assessed the sensitivity of PC9 and HCC827 cells to gefitinib by CCK-8 assays. As shown in Fig. 2D, both PC9 and HCC827 cells exerted a lower sensitivity to gefitinib when LOC554202 and miR-31 were overexpressed, respectively. Together, these data demonstrated that LOC554202 and miR-31 decreased gefitinib sensitivity of NSCLC cells to gefitinib.

### Elevated expression of miR-31 promotes proliferation *in vitro* and* in vivo*

**Figure 3:**
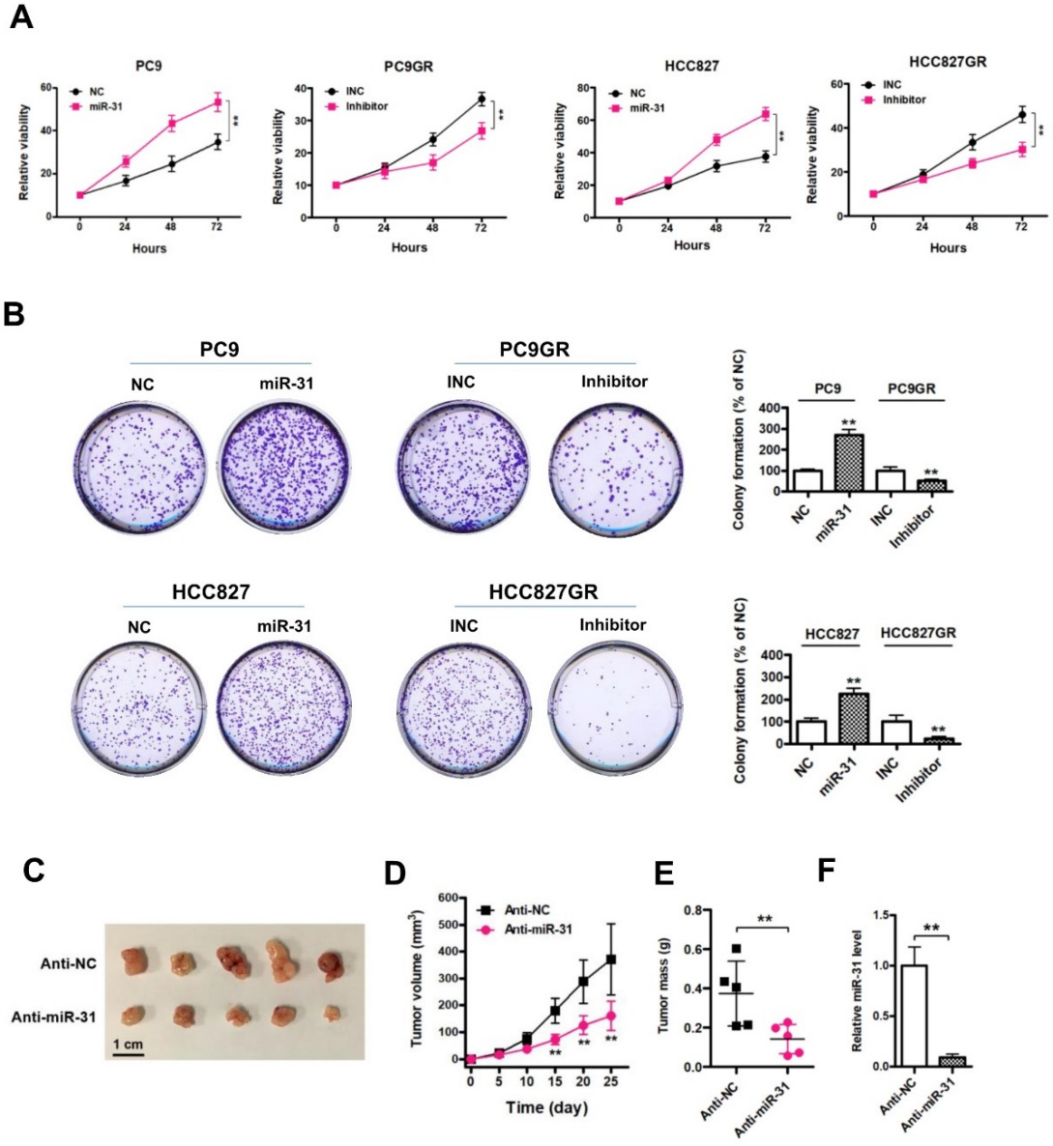
** Elevated expression of miR-31 promotes proliferation *in vitro* and* in vivo.* A**. Cell proliferation was measured by CCK-8 assays when miR-31 was transfected in PC9 and HCC827 cells, and knocked down in PC9GR and HCC827GR cells by inhibitors, respectively. **P < 0.01. Experiments were performed in triplicates. Data are represented as mean ± SD. **B**. Colony-formation assays were performed when miR-31 was overexpressed in PC9 and HCC827 cells, and knocked down in PC9GR and HCC827GR cells inhibitors, respectively. **P < 0.01. Experiments were performed in triplicates. Data are represented as mean ± SD. **C**. Images of isolated xenograft tumors from control (Anti-NC) and miR-31 knockdown (Anti-miR-31) groups. **D**. Growth curves of Anti-NC and miR-31 knocked down PC9GR cells *in vivo*. **P < 0.01. Data are represented as mean ± SD. **E**. Comparison of average xenograft tumor weights from control (Anti-NC) and miR-31 knockdown (Anti-miR-31) groups. n = 5; **P < 0.01.** F**. Real-time PCR analysis of miR-31 expression levels in xenograft tumor tissues from control (Anti-NC) and miR-31 knockdown (Anti-miR-31) groups. n = 5; **P < 0.01. Data are represented as mean ± SD.

To explore the role of miR-31 in the regulation of acquired resistance to gefitinib, we performed CCK-8 assays in NSCLC cells. First, we observed that elevated expression of miR-31 mimics increased PC9 and HCC827 cell proliferation and colony-formation abilities, whereas silencing of miR-31 by transfecting miR-31 inhibitors inhibited PC9GR and HCC827GR cell proliferation and clonogenic growth* in vitro* (Fig. 3A&B; **P < 0.01). Furthermore, we established *in vivo* models to further validate the role of miR-31 in PC9GR cell proliferation by injecting miR-31 knockdown (Anti-miR-31) and control (Anti-NC) PC9GR cells into nude mice. Most impressively, silence of miR-31 in PC9GR cells resulted in a remarkable tumor shrink *in vivo* (Fig. 3C). The volume and mass of excised xenograft tumors were both significantly decreased when miR-31 was interfered (Fig. 3D&E; **P< 0.01). And the knockdown of miR-31 in xenograft tumors was also verified by Real-time PCR assays (Fig. 3F; **P < 0.01). Put together, our results demonstrated that miR-31 promoted gefitinib-resistant NSCLC cells proliferation *in vitro* and *in vivo*.

### LOC554202 and miR-31 are upregulated in NSCLC patients with acquired resistance to gefitinib

**Figure 4:**
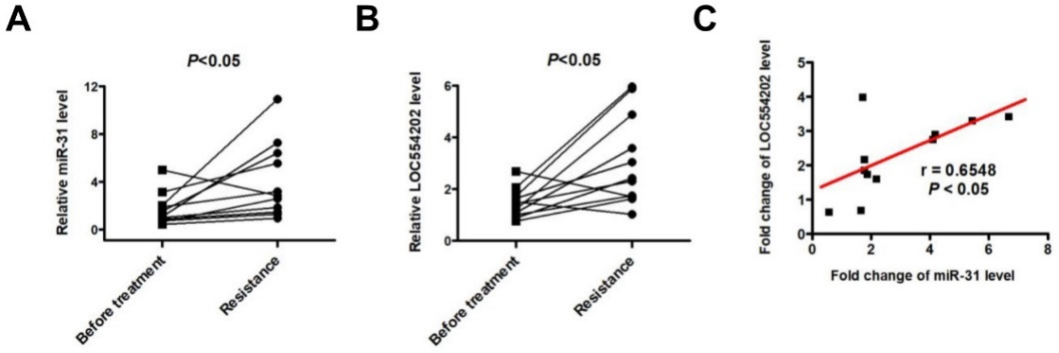
** LOC554202 and miR-31 are upregulated in NSCLC patients with acquired resistance to gefitinib.** Real-time PCR analysis of plasma miR-31 (**A**) and LOC554202 (**B**) in before (Before treatment) and after treatment (Resistance) with gefitinib from 11 NSCLC patients acquiring resistance to gefitinib. *P < 0.05. **C**. The spearman rank correlation analysis was performed to analyze the correlation between LOC554202 and miR-31 in 11 NSCLC patients with acquired resistance to gefitinib (r = 0.6548; *P* < 0.05).

To detect the expression of LOC554202 and miR-31 levels in NSCLC patients treated with EGFR-TKI, we collected 11 advanced NSCLC patients with acquired resistance to gefitinib and total RNA was isolated from plasma of 11 patients at the time points of before and after gefitinib treatment. As shown in Fig. 4A&B, both LOC554202 and miR-31 levels were significantly increased in NSCLC patients acquiring resistance to gefitinib (*P < 0.05). More importantly, the expression of LOC554202 was positively correlated with the expression of miR-31 in NSCLC patients with acquired resistance to gefitinib (r = 0.6548; *P<0.05), which is consistent with our results in Fig. 2B&C.

### RASA1 and FIH-1 are direct targets of miR-31

**Figure 5:**
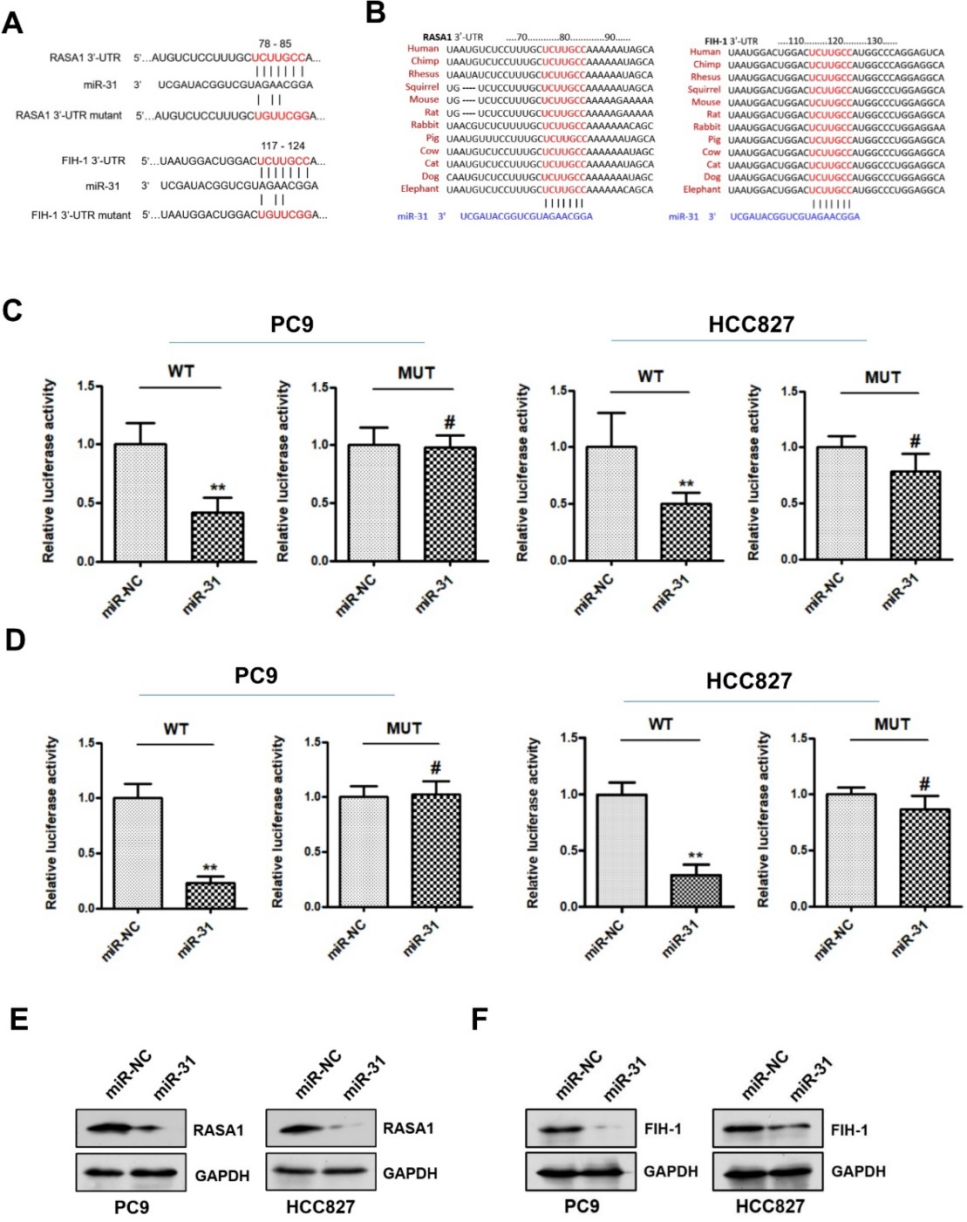
** RASA1 and FIH-1 are direct targets of miR-31. A.** The predicted binding sites of miR-31 in 3'-UTR of RASA1 and FIH-1 mRNA. **B**. DNA sequence of miR-31 binding-sites in 3'-UTR of RASAI and FIH-1 mRNA were highly conserved in different species. **C**. Relative luciferase activities of PC9 and HCC827 cells co-transfected with wild-type (WT) or mutated (MUT) reporter constructs containing the 3'-UTR of RASA1 and miR-31 mimics or negative control mimics (miR-NC). **D**. Relative luciferase activities of PC9 and HCC827 cells co-transfected with wild-type (WT) or mutated (MUT) reporter constructs containing the 3'-UTR of FIH-1 and miR-31 mimics or negative control mimics (miR-NC). Luciferase activity was normalized to β-gal activity. **P < 0.01; ^#^*P* > 0.05. Data are represented as mean ± SD. Western blot analysis of RASA1 (**E**) and FIH-1 (**F**) were detected in PC9 and HCC827 cells transfected with miR-31 or miR-NC mimics. GAPDH was used as endogenous loading control.

To explore the potential mechanism underlying miR-31 in acquired resistance to gefitinib in patients with NSCLC, TargetScan database (www.targetscan.org) was used to predict the potential target genes of miR-31. RASAI and FIH-1 were predicted bioinformatically as putative targets of miR-31. As shown in Fig. 5A&B, the DNA sequence of miR-31 binding-sites in 3'-UTR (3'-untranslated regions) of RASAI and FIH-1 were highly conserved in different species (including human, chimp, rhesus, squirrel, mouse, rat, rabbit, pig, cow, cat, dog and elephant). To determine whether RASAI and FIH-1 are direct target genes of miR-31, luciferase reporter gene assays were performed in PC9 and HCC827 cells. In Fig. 5C&D, PC9 and HCC827 cells transfected with miR-31 mimics showed a markedly decreased luciferase activity in contrast to those transfected with negative control mimics (**P < 0.01). However, this reduction of luciferase activity was abolished when the DNA sequence of miR-31 binding sites in 3'-UTR of RASAI and FIH-1 were mutated, respectively (MUT; ^#^*P* > 0.05). Furthermore, the RASA1 and FIH-1 protein expression levels were also examined in PC9 and HCC827 transfected with miR-31 and NC mimics. Indeed, Western blot assays showed that the endogenous RASA1 (Fig. 5E) and FIH-1 (Fig. 5F) protein expression levels were obviously downregulated when miR-31 was overexpressed in PC9 and HCC827. Collectively, these data demonstrated that miR-31 could negatively regulate RASAI and FIH-1 expression in PC9 and HCC827 cells by targeting their 3'-UTR directly.

### MiR-31 activates RAF-MEK-ERK and PI3K-AKT signaling pathways through repressing RASA1 and FIH-1

**Figure 6:**
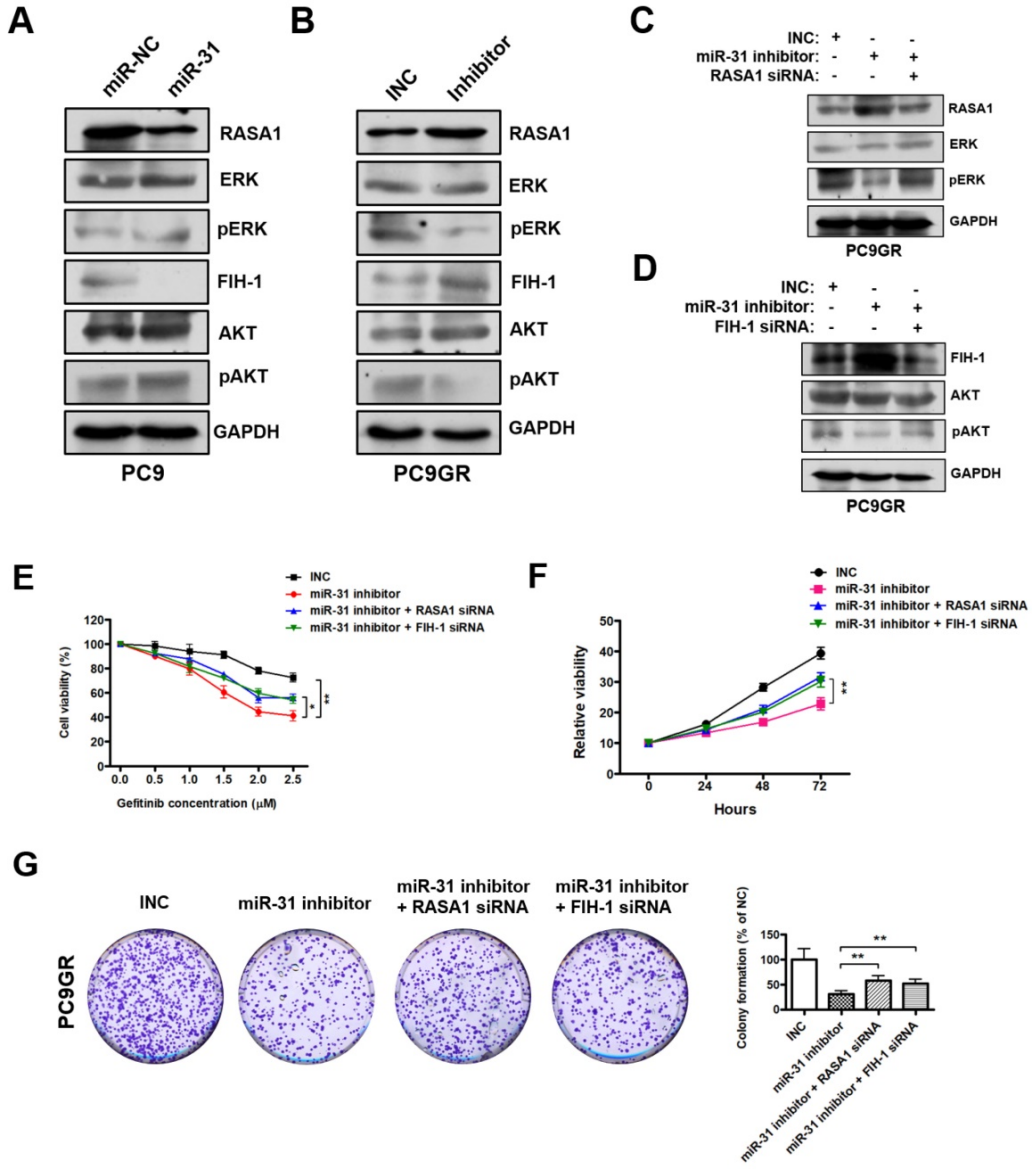
** MiR-31 activates RAF-MEK-ERK and PI3K-AKT signaling pathways through repressing RASA1 and FIH-1. A**. Western blot analysis of RASA1, ERK, pERK, FIH-1, AKT and pAKT in PC9 cells transfected with miR-31 or miR-NC mimics. **B**. Western blot analysis of RASA1, ERK, pERK, FIH-1, AKT and pAKT in PC9GR cells transfected with INC and miR-31-inhibitor mimics (Right panel). **C**. Western blot analysis of RASA1, ERK and pERK protein levels in PC9GR cells transfected with INC, miR-31-inhibitor, miR-31-inhibitor + RASA1 siRNA mimics. **D**. Western blot analysis of FIH-1, AKT and pAKT protein levels in PC9GR cells transfected with INC, miR-31-inhibitor, miR-31-inhibitor + FIH-1 siRNA mimics. GAPDH was used as endogenous loading control. **E**. The proliferation ability of INC, miR-31 inhibitor, miR-31 inhibitor + RASA1 siRNA and miR-31 inhibitor + FIH-1 siRNA-treated PC9GR cells in response to different concentrations of gefitinib. *P < 0.05, **P < 0.01. Data are represented as mean ± SD. **F**. CCK-8 assays were performed to determine the proliferation ability of PC9GR cells treated with INC, miR-31 inhibitor, miR-31 inhibitor + RASA1 siRNA and miR-31 inhibitor + FIH-1 siRNA, respectively. **P < 0.01. Data are represented as mean ± SD. **G**. Colony-formation assays were performed when PC9GR cells were treated with INC, miR-31 inhibitor, miR-31 inhibitor + RASA1 siRNA and miR-31 inhibitor + FIH-1 siRNA, respectively. **P < 0.01. Data are represented as mean ± SD.

RASA1 and FIH-1 play important roles in many cellular and biological processes, including anti-cancer drugs resistance and proliferation regulation. Previous reports imply that RASA1 and FIH-1 may act as negative regulators in RAF-MEK-ERK and PI3K-AKT signaling pathways^32^, ^33^. Therefore, we examined whether miR-31 regulates the key signaling molecules of RAF-MEK-ERK and PI3K-AKT pathways by Western blot. As shown in Fig. 6A, pERK (phosphorylated ERK) and pAKT (phosphorylated AKT) levels were dramatically increased in PC9 cells transfected with miR-31, whereas miR-31 knockdown by inhibitors dramatically decreased the pERK and pAKT levels in PC9GR cells (Fig. 6B). To investigate whether miR-31 activates RAF-MEK-ERK and PI3K-AKT signaling by targeting RASA1 and FIH-1, rescue assays were performed in PC9GR cells. Surprisingly, in Fig. 6C, RASA1 knockdown by siRNA could rescue the miR-31-inhihbitors mediated downregulation of pERK in PC9GR cells. Similarly, FIH-1 knockdown could also rescue the miR-31-inhihbitors mediated downregulation of pAKT in PC9GR cells (Fig. 6D).

To examine whether RASA1 and FIH-1 play important roles in miR-31-mediated acquired resistance to gefitinib of NSCLC cells, we silenced RASA1 and FIH-1 expression in miR-31-knockdown PC9GR cells and then assessed the sensitivity of PC9GR cells to gefitinib. As shown in Fig. 6E, PC9GR cells exerted a higher sensitivity to gefitinib when miR-31 was knocked down (**P < 0.01). However, RASA1 or FIH-1 knockdown could partially rescue the miR-31 inhibitors-mediated increase in sensitivity to gefitinib (*P < 0.05). In addition, CCK-8 and colony-formation assays showed that RASA1 or FIH-1 knockdown could, at least partially, rescue the miR-31 inhibitors-mediated reduced proliferation and colony-formation phenotype of PC9GR cells (Fig. 6F&G, **P < 0.01), demonstrating that miR-31 could lead to decreased gefitinib sensitivity of PC9GR cells by targeting RASA1 and FIH-1. Together, these data proved that miR-31, at least partially activated RAF-MEK-ERK and PI3K-AKT signaling pathways by directly repressing RASA1 and FIH-1 in NSCLC cells.

## Discussion

LncRNA and miRNAs were frequently aberrantly expressed in a variety of human tumors. They are usually characterized as tumor suppressors or oncogenes in regulating carcinogenesis, tumor progression and resistance to therapy 22, 24. Previous studies have demonstrated that LOC554202 (2166 bp transcript on chromosome 9p21.3), as the host gene of miR-31, affected the expression of miR-31 and played important roles in the progression and metastasis of cancer^34^, ^35^. However, little is known about the correlation between LOC554202 and miR-31 expression levels, and their biological functions in cancer biology, especially in NSCLC.

Ma *et al*. elucidated the relationship between LOC554202 and miR-31 in chordoma^34^. They found that up-regulation of LOC554202 was associated with a decreased level of miR-31 in chordoma tissues. Moreover, silencing of LOC554202 or upregulation of miR-31 could repress the proliferation and migration/ invasion of chordoma cells. In laryngeal squamous cell carcinoma (LSCC), Yang *et al*. observed that the expression of miR-31 was negatively correlated with the expression of LOC55420^35^. Overexpression of LOC554202 increased Hep-2 cell proliferation, cell cycle and invasion and overexpression of miR-31 inhibited Hep-2 cell proliferation, cell cycle and invasion. Consequently, these studies suggested that miR-31 was a tumor repressor in tumor. Contrary to these conclusions, Xi and colleagues reported that over-expression of miR-31, as a oncomir, significantly enhanced proliferation and tumorigenicity of lung cancer cells whereas downregulation of miR-31 repressed proliferation of lung cancer cells^36^. We are still a bit confused about the relationship between LOC554202 and miR-31, and also their biological functions in tumors. These studies indicated that LOC554202 and miR-31 may play different roles in different tumors.

Up to date, there are very few reports about the relationship between LOC554202 and miR-31 expression levels in NSCLC with acquired resistance to gefitinib. Therefore, we investigated the correlation between LOC554202 and miR-31, and their functions in regulation of EGFR TKI-resistant NSCLC. We observed that LOC554202 and miR-31 levels were significantly increased in NSCLC patients acquiring resistance to gefitinib. The expression of LOC554202 was positively correlated with the expression of miR-31 in NSCLC patients with acquired resistance to gefitinib. More importantly, we found that LOC554202 and miR-31 promoted proliferation and clonogenic growth of gefitinib-resistant NSCLC cells *in vitro* or* in vivo*. Further data demonstrated that LOC554202 and miR-31 decreased gefitinib sensitivity of NSCLC cells to gefitinib. These results, for the first time, suggested that LOC554202 and miR-31 both act as oncogenes in regulation of EGFR TKI-resistance in NSCLC.

MiR-31, encoded by a single genomic locus, is expressed in variety of human cancers and may represent one of the most potential targets for clinical treatment^37^. In previous natural language processing (NLP) analysis, we suggested that miR-31 may be associated with initiation, progression and resistance of lung cancer by regulating MAPK signaling, p53 signaling, Toll-like receptor signaling pathways, as well as others^38^. Generally, miR-31 functions as oncogenes in most cancers^39^. However, miR-31 has also been characterized as tumor suppressor or metastasis inhibitor in other cancers^40^. Taken together, miR-31 may play different cancer-related roles in different tumor types and genetic background. Therefore, it's necessary to carry out comprehensive and systematic studies to better understand the mechanisms of miR-31 leading to EGFR TKI resistance of NSCLC. We first identified RAS p21 GTPase activating protein 1 (RASA1) and FIH-1 as the direct targets of miR-31 by luciferase reporter assays. MiR-31 could directly bind to the 3'-UTR of RASA1 and FIH-1, and inhibit upstream luciferase activity. However, this inhibition of luciferase activity was dramatically abolished when the DNA sequence of miR-31 binding sites in 3'-UTR of RASAI and FIH-1 were mutated. In addition, the endogenous RASA1 and FIH-1 protein expression levels were reduced when miR-31 was overexpressed in NSCLC cells. RASA1 is a Ras GTPase-activating protein that functions as a general inhibitor of RAS functions by transferring the GTPase activity of normal RAS proteins to inactive GDP-bound form of RAS, thus resulting in aberrant signaling through the RAF-MEK-ERK and PI3K-AKT signaling pathways^32^. FIH-1 (Hypoxia inducible factor 1 subunit alpha inhibitor) could interact with HIF-1α and act as a negative regulator of PI3K/AKT signaling pathway^33^. According to previous reports, activation of RAF-MEK-ERK and PI3K-AKT signaling pathways play important roles in the resistance to EGFR-TKIs of NSCLC, such as gefitinib and erlotinib^4^, ^5^. In this study, we investigated whether miR-31 regulates the key signaling molecules of ERK and AKT pathways *in vitro*. We found the key molecules of RAF-MEK-ERK and PI3K-AKT pathways pERK (phosphorylated ERK) and pAKT (phosphorylated AKT) levels were dramatically increased in PC9 cells overexpressing miR-31, whereas miR-31 knockdown dramatically decreased the pERK and pAKT levels in PC9GR cells. Rescue assays suggested that RASA1 or FIH-1 knockdown could rescue the miR-31-inhihbitors mediated downregulation of pERK and pAKT in PC9GR cells, respectively. These results suggested that miR-31 could activate ERK and AKT signaling by repressing RASA1 and FIH-1, and thereby upregulation of miR-31 in NSCLC may activate RAF-MEK-ERK and PI3K-AKT signaling pathways by suppressing the expression of RASA1 and FIH-1, and thus resulting in EGFR TKI-resistance in NSCLC.

In summary, our study, for the first time, investigated the expression of LOC554202 and miR-31 in NSCLC with acquired gefitinib resistance. Functional and mechanistic studies suggested that overexpression of miR-31 promoted NSCLC cells proliferation and clonogenic growth *in vitro* and *in vivo* and reduced the sensitivity of NSCLC cells to gefitinib. More importantly, upregulation of miR-31 in NSCLC may regulate RAF-MEK-ERK and PI3K-AKT signaling pathways by directly targeting RASA1 and FIH-1, and thus contributing to the acquired gefitinib resistance. This study may provide new insight into mechanisms of acquired resistance to EGFR TKI in NSCLC and a potential target for ant-cancer drug treatment.

## Supplementary Material

Supplementary figures and tables.
