# Supplementary material for: Long non-coding RNA LOC554202 promotes acquired gefitinib resistance in non-small cell lung cancer through upregulating miR-31 expression
Source: J Cancer. 2019 Oct 15;10(24):6003–13. doi: 10.7150/jca.35097 (PMC6856583; doi:10.7150/jca.35097)
Supplement: Supplementary file 1 — Supplementary figures and tables. [file jcav10p6003s1.pdf]

Supplementary Table 1. Basic clinical parameters of the enrolled patients

No.	Gender	Age (year)	Location	Pathological type	EGFR mutation	TNM stage	Treatmet response	PFS (month)
1	Male	43	Left	Adenocarcinoma	L858R	Ⅳ	PR	11
2	Male	67	Left	Adenocarcinoma	L858R	Ⅳ	SD	20
3	Female	57	Right	Adenocarcinoma	19 del	Ⅳ	PR	16
4	Female	46	Left	Adenocarcinoma	19 del	Ⅳ	SD	32
5	Male	41	Left	Adenocarcinoma	19 del	Ⅳ	PR	12
6	Male	66	Right	Adenocarcinoma	L858R	Ⅳ	SD	6
7	Female	66	Right	Adenocarcinoma	19 del	ⅢB	PR	8
8	Male	74	Left	Adenocarcinoma	19 del	ⅢB	SD	4
9	Male	80	Right	Adenocarcinoma	L858R	Ⅳ	SD	9
10	Male	59	Left	Adenocarcinoma	19 del	ⅢB	SD	12
11	Male	69	Left	Adenocarcinoma	L858R	Ⅳ	PD	1
